# Influence of main forcing affecting the Tagus turbid plume under high river discharges using MODIS imagery

**DOI:** 10.1371/journal.pone.0187036

**Published:** 2017-10-26

**Authors:** D. Fernández-Nóvoa, M. Gómez-Gesteira, R. Mendes, M. deCastro, N. Vaz, J. M. Dias

**Affiliations:** 1 EPHYSLAB, Environmental PHYsics LABoratory, Facultad de Ciencias, Universidad de Vigo, Ourense, Spain; 2 CESAM, Physics Department, University of Aveiro, Aveiro, Portugal; Centro de Investigacion Cientifica y de Educacion Superior de Ensenada Division de Fisica Aplicada, MEXICO

## Abstract

The role of river discharge, wind and tide on the extension and variability of the Tagus River plume was analyzed from 2003 to 2015. This study was performed combining daily images obtained from the Moderate Resolution Imaging Spectroradiometer (MODIS) sensor located onboard the Aqua and Terra satellites. Composites were generated by averaging pixels with the same forcing conditions. River discharge shows a strong relation with the extension of the Tagus plume. The plume grows with the increasing river discharge and express a two day lag caused by the long residence time of water within the estuary. The Tagus turbid plume was found to be smaller under northerly and easterly winds, than under southerly and westerly winds. It is suggested that upwelling favoring winds provoke the offshore movement of the plume material with a rapidly decrease in turbidity values whereas downwelling favoring winds retain plume material in the north coast close to the Tagus mouth. Eastern cross-shore (oceanward) winds spread the plume seaward and to the north following the coast geometry, whereas western cross-shore (landward) winds keep the plume material in both alongshore directions occupying a large part of the area enclosed by the bay. Low tides produce larger and more turbid plumes than high tides. In terms of fortnightly periodicity, the maximum plume extension corresponding to the highest turbidity is observed during and after spring tides. Minimum plume extension associated with the lowest turbidity occurs during and after neap tides.

## Introduction

Turbid plumes are formed near river mouths in adjacent coastal areas by the transport of materials discharged by the river into the sea. Turbid plumes play a major role in the physical, chemical and biological evolution of coastal waters since they transport sediments, nutrients, microscopic organisms, contaminants and fresh water in an area bursting with economic activity [1]. It is important to characterize the influence of river plumes to better understand their temporal and spatial variability. Although each river plume behaves differently, some features are common. The discharge of rivers into the ocean usually produces an anticyclonic gyre that traps a coastal current flowing in the direction of the Kelvin wave propagation. In addition, plume displacement is dependent on the topography and momentum advection [2]. Upwelling winds tend to move the plume seaward due to the offshore Ekman transport, whereas downwelling winds have the opposite effect, compressing the plume against the coast as a result of an onshore transport [3]. Tidal forcing also constrains the plume propagation. The high phase of the semidiurnal cycle usually limits plume formation [4]. Moreover, the spring-neap tidal cycle can generate changes in the concentration of suspended sediments within the plume, with maximum turbidity values during spring tides [5, 6].

Ocean color satellite imagery was successfully used to observe the behavior of river plumes under different situations [7–9]. For example, [7] mapped the main features of Mississippi plume, [8] evaluated the seasonal variability of river plumes along the central Chilean coast and [9] monitored the river plume during heavy rainfall events, all using MODIS imagery. Remote sensing imagery of the Iberian Peninsula also identified the main processes controlling the turbid plume development of the Douro and Ebro Rivers [10, 11]. Synoptic maps of these plumes were constructed to evaluate their mean state under the main forcing affecting them. Differentiating turbid waters associated with river discharge from surrounding waters is rendered possible due to their different optical properties detected by ocean color imagery [12–14]. These studies highlight that river plumes usually present a great amount of suspended material resulting in a strong signal detected by the satellite sensor whereas the ocean clear waters have negligible contributions. Turbidity is used to assess the quality of water since it can affect light attenuation, nutrient dynamics, transport of pollutants and phytoplankton productivity [15]. Tracking turbidity also helps understand the distribution of total suspended sediments and coastal erosion.

The Tagus estuary is the main source of sediments for the coastal area and plays a key role in erosion and sedimentation [5]. The Tagus plume controls the light and nutrient availability in the affected area, regulates the production of phytoplankton and alters the retention and dispersion of larvae [6]. Therefore, the coastal productivity and biogeochemistry, as well as the ambient coastal circulation are dependent on the evolution of the Tagus River plume.

The main forcing factors controlling the Tagus River plume are the river discharge, wind and tide according to [4]. Previous research on the response of the Tagus plume to these processes were carried out through numerical modeling, specifically on the effect of river runoff and upwelling winds during the winter of 2007 [4] and the semi-diurnal and spring-neap tidal influence during the 2008–2009 period [6]. [5] revealed the strong influence of the spring-neap tidal cycle on the dimension of the Tagus plume through remote sensing imagery. During the 2003–2005 period, the authors observed a large and highly turbid plume controlled by the spring tide using normalized water-leaving radiance at the 551 nm wavelength (nLw551).

The present study aims to characterize and understand the spatial and time variability of the Tagus turbid plume in relation to the principal driving mechanisms (river discharge, wind stress and tides). Moderate Resolution Imaging Spectroradiometer (MODIS) data will be used to monitor the plume development for each synoptic situation over a period ranging from 2003 to 2015. Radiance data from similar forcing situations were averaged in order to form robust composite views to analyze plume behavior under the effect of different drivers. The effect of overlapping semi-diurnal and fortnightly tidal cycles on plumes formed through rivers discharging in large estuaries has not been previously investigated from this point of view. Moreover, large estuaries generate a longer water residence time, which is an important component on plume behavior and dispersion when compared to rivers directly flowing into the ocean. Understanding of the Tagus plume propagation and development can also prepare for droughts and/or especially flooding events.

## Study area, databases and methodology

### Study area

The Tagus River originates in Teruel (Spain) and flows into the Atlantic Ocean near Lisbon (Portugal). It is the longest Iberian river (1008 km) with a basin of approximately 81,000 km^2^. The average river flow is 331 m^3^s^-1^ [16], with maximum monthly discharges averaging ~ 2200 m^3^s^-1^ [5]. The Trancão, Sorraia, and Ribeira de Coina Rivers flowing into the estuary, with the Tagus, have a very low runoff with a minimal influence on the generation of the Tagus plume.

**Fig 1:**
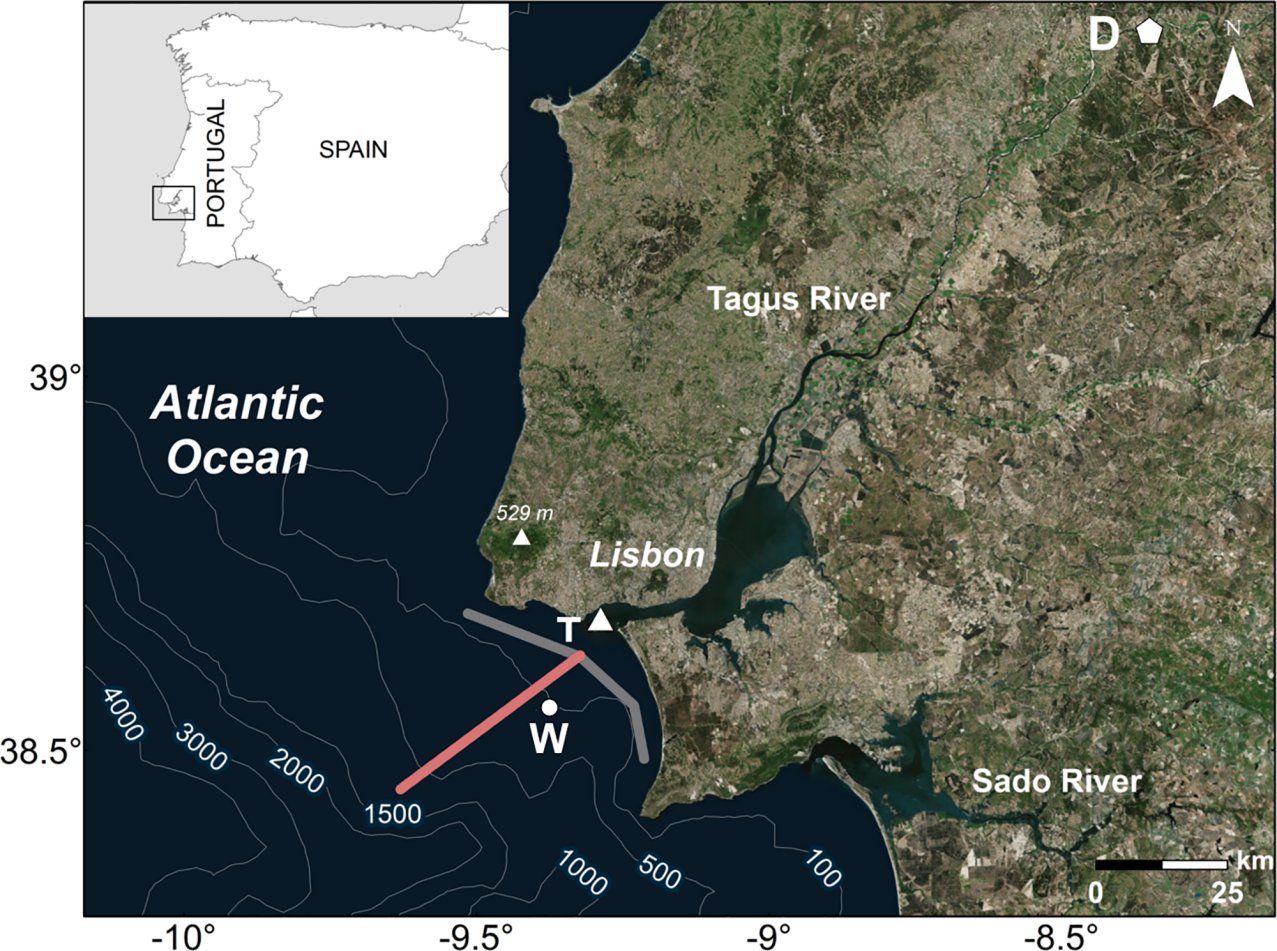
Localization of the study area. Tagus River mouth is located between capes Espichel and Raso (Tagus ROFI area). D marks the position of the Almourol station where river discharge data was obtained. W indicates the point where wind data were acquired. T represents the area of Cascais where tidal data was predicted. Grey and pink lines mark the alongshore and cross-shore transects used to analyze turbidity. Service Layer Credits Source: Esri, DigitalGlobe, GeoEye, icubed, Earthstar, Geographics, CENS/Airbus DS, USDA, USGS, AEX, Getmapping, Aerogrid, IGN, IGP, swisstopo, and the GIS User Community.

The Tagus estuary occupies a volume of 1900×10^6^ m^3^ and a surface area of 320 km^2^ [5]. The estuary joins the Atlantic Ocean through a 30 m deep, 2 km wide and 12 km long channel [17], forming a NE-SW oriented thalweg. This makes the Tagus estuary the most important of the Iberian Peninsula and one of the largest of the European west coast (Fig 1). Tides are primary semi-diurnal but with fortnightly periodicity. They present an average amplitude of 2.4 m at the river mouth, and range between 0.9 m at neap tide and 4.1 m at spring tide [18].

Flow stratification is regulated by water exchange, and is strongly influenced by estuarine circulation and outflow as found in modelling [4, 17] and observational studies [19] held in the regions near the Tagus mouth. Model results obtained using a 3D hydrodynamic model [17] reveal that the estuary mouth exhibits tidal pumping and chaotic advection which contribute to generation of mixing and of two vortexes that affect circulation [17, 20], turning the transport of solute and particulate material a very complex issue. Moreover, these complex features also generate preferential transport pathways within the estuary, which can change in short time scales [20].

The spreading of Tagus plume through the coast is mainly controlled by tides and wind forcing [4]. The effect of the wind depends on vertical stratification, which in turn relies on the heat exchange with the atmosphere and on salinity advection, which is highly affected by the estuarine outflow [4]. Tides affect circulation in the whole estuarine region, extending 50 km inward from the estuary mouth, with a maximum induced current speed of 2 ms^-1^ under spring tides [18]. Flooding leads to a net export of sediments and they persist longer than the ebbs [20].

All these results rely on the implementation of circulation and transport models to the area comprising the Tagus Estuary and the near coastal waters. However, all these implementations have associated an amount of uncertainty, which can produce inaccurate conclusions. Moreover, observational programs that uses standard oceanographic probes are often limited both spatially and temporally. Therefore, a step forward is assumed in this study by using satellite data in order to create a methodology to study estuarine plume propagation. This methodology allows the continuous study of the Tagus plume and can be a useful tool in future modelling studies that integrate high resolution models and satellite data.

The coast is S-N oriented except near the river mouth, which is oriented SE to NW. Plume dynamics are modified by coastline geometry, with a trajectory near the shore [4]. In addition, a narrow shelf may perturb coastal currents (with typical surface velocity values ~1 ms^-1^ near the river mouth) and therefore affects the development of the turbid plume [19]. The estuarine outflow is protected from the waves, which have a dominant NW direction, by the configuration and orientation of the coastline with only the southern part of the estuarine channel exposed to the swells [21].

There are important topographic features that should be evaluated because they affect the development of the Tagus plume (Fig 1). The shelf area situated between the Espichel and Raso capes (38.41°N-38.78°N latitude and 9.22°W-9.51°W longitude) is thereby labeled the Tagus ROFI (Region Of Freshwater Influence). The Sintra Mountains (north of Lisbon) control in part the wind patterns in the ROFI and submarine canyons will modify the circulation of seawater on the shelf.

The study area covers a region comprised between 38.3°N-39°N latitude and 8.75°W-9.8°W longitude (Fig 1), with special attention to the region included in the Tagus ROFI. This region only takes into account the effect of the Tagus plume minimizing the influence of other small river plumes (principally the Sado River plume) and/or overestimations of turbidity caused by other processes as upwelling blooms or re-suspension [22] on the Tagus plume area.

### Databases

#### Ocean color imagery

Radiance data in the study area was obtained from MODIS located onboard the Aqua and Terra satellites over the period of 2003 to 2015. Radiance data was retrieved from the NASA Ocean Color web site (http://oceancolor.gsfc.nasa.gov), and were processed and distributed by the Ocean Biology Processing Group (OBPC) at the Goddard Space Flight Center.

L1A files were processed to L1B files by the SeaDAS software (SeaWIFS Data Analysis System, version 6.4, [23]), following standard procedures recommended for raw data files processing. L1B files were then converted to L2 files by applying a methodology similar to that provided by [24]. As main processing features, clouds with a threshold albedo of 0.018 were masked and the high light masks were disabled. Moreover, stray light masks with a 3 x 3 array were applied around land and clouds and the unrealistic values of remote sensing reflectance (negative values and extremely high values, which could be caused by sensor saturation) were discarded. The produced data were processed at a resolution of 250 m for the nLw645 band, improving the resolution used in previous studies [10, 11, 24]. River plumes along the Iberian Peninsula with extension and turbidity similar to the Tagus River one, were successfully performed with 500m of resolution [10, 11]. In addition, [24] pointed out that a resolution of 500m was enough for the Mississippi, Yangtze and Amazon Rivers and the Chesapeake Bay.

MODIS allows working with several normalized water-leaving radiances (nLw). Some elements need consideration when selecting the most suitable band to study the Tagus plume. First, a high spatial resolution is needed, thus only the nLw469, 555 and 645 bands are acceptable for a 500 m spatial resolution (Table 1). The best coverage (in number of days) is provided by bands 488, 531, 547 and 555 and the best correlation with the river discharge is obtained through bands 645, 667 and 678 (Table 1). Overall, nLw555 and 645 seem to be the appropriate bands to utilize. However, the nLw645 band was chosen because it has a lower water penetration depth [15], avoids the interference from the shallow areas seafloor and reduces the influence of turbidity caused by upwelling or re-suspension. In addition, the nLw645 band allows a maximum resolution of 250 m. Daily nLw645 band data obtained from each satellite were interpolated into a regular mesh of 0.0025° × 0.0025°.

**Table 1:** Comparison of the different available bands for the Aqua and Terra satellites.

Band (nm)	MODIS_Aqua (Terra) satellite sensor				
	Spatial resolution (m)	p (%)	r		
			lag 0	lag 1	lag 2
412	1000	20 (24)	0.01 (0.01)	0.01 (-0.01)	0.03 (0.02)
443	1000	28 (29)	0.14 (0.13)	0.13 (0.12)	0.11(0.10)
469	500	30 (30)	0.23 (0.20)	0.23 (0.19)	0.21 (0.17)
488	1000	31 (31)	0.29 (0.28)	0.28 (0.28)	0.27 (0.27)
531	1000	31 (31)	0.38 (0.38)	0.38 (0.38)	0.37 (0.37)
547	1000	31 (31)	0.40 (0.40)	0.40 (0.40)	0.40 (0.40)
555	500	31 (31)	0.41 (0.41)	0.41 (0.41)	0.42 (0.41)
645	250	20 (22)	0.56 (0.52)	0.56 (0.53)	0.57 (0.54)
667	1000	17 (19)	0.59 (0.54)	0.59 (0.55)	0.59 (0.55)
678	1000	21 (25)	0.57 (0.50)	0.58 (0.50)	0.57 (0.51)

The parameters considered were: the spatial resolution, percentage of available days (p) (daily images were considered valid if more than 70% of the pixels were available), and the correlation between nLw and river discharge (r) under different lags. For all parameters, the first number corresponds to the Aqua satellite and the number in brackets relates to the Terra satellite.

#### River discharge data

**Fig 2:**
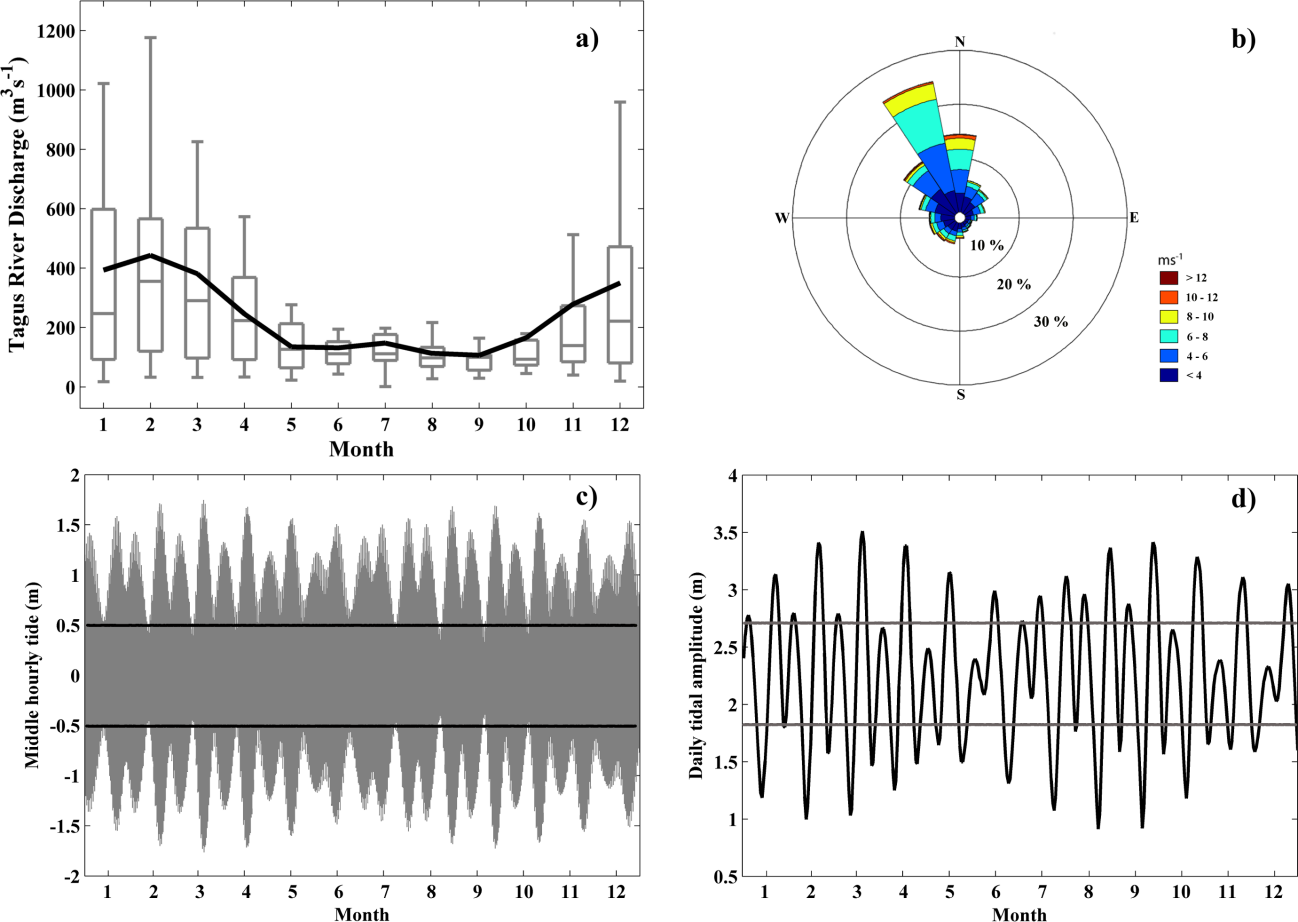
(a) Annual hydrologic cycle for Tagus River discharge (m^3^s^-1^) obtained from 2003 to 2015. Solid black line: monthly average; the line inside each box indicates the median; lower and upper whiskers: minimum and maximum, respectively; lower and upper box limits: first and third quartiles, respectively, (b) Wind rose (ms^-1^) of the Tagus ROFI during the period of 2003 to 2015, (c) Surface elevation of seawater at Cascais for 2007. Solid lines represent the limits of high and low tide and, (d) Daily tidal amplitude at Cascais for 2007. The solid lines indicate the limits of spring and neap tides.

Daily runoff data were obtained from the SNIRH (“Sistema Nacional de Informação de Recursos Hídricos”) database (www.snirh.pt) at Almourol hydrometric Station (Fig 1) from 2003 to 2015. The river flow presents seasonal variations, with high discharges in winter reaching a maximum in February with an average monthly value of ~ 450 m^3^s^-1^ and low discharges in summer showing a minimum in September with a mean monthly value of ~ 100 m^3^s^-1^ (Fig 2A). The mean daily river discharge is 249 m^3^s^-1^, with a minimum daily value of 1 m^3^s^-1^ and a maximum of 5010 m^3^s^-1^.

#### Wind data

Wind data were obtained from the NOAA’s National Operational Model Archive and Distribution System (NOMADS), which is maintained at NOAA’s National Climatic Data Center (NCDC) [25]. The database Climate Forecast System Reanalysis (CFSR) was developed by NOAA’s National Centers for Environmental Prediction (NCEP) (http://rda.ucar.edu/pub/cfsr.html). The wind data is available at 6 hour intervals and possess a 0.3°×0.3° spatial resolution, covering the atmosphere, ocean, sea ice and land. The CFSR database was selected for its high spatial and time resolution. [26] demonstrated the CFSR data are correlated with buoy measurement data taken along the Atlantic Iberian Coast so it was used to investigate river plumes in this area [10, 11]. Wind data at a reference height of 10 m were daily averaged for a location near the river mouth within the Tagus ROFI area (Fig 1).

Northerly winds are prevalent along the Western Iberian coast with less important southerly winds [27]. In contrast, the Tagus ROFI area was principally subjected to predominant north-westerly winds, followed by northerly and north-easterly winds, with velocities ranging from 4 to 10 ms^-1^ (Fig 2B). The different wind patterns are related to the particular orientation of the coastal area and to the attenuation of northerly winds by the Sintra Mountains.

We considered both alongshore and cross-shore winds. Alongshore winds include northern and southern alongshore directions whereas cross-shore winds include eastern and western cross-shore directions (according with the meteorological convention that considers the direction from which the wind blows). To avoid calm intervals, only winds with average velocities >2 ms^-1^ lasting for a 2 day period before the date of recording were included. Finally, we allowed a deviation of ±30° for the wind directions. Note the wind directions were rotated counter-clockwise to follow the shoreline. However, for sake of clarity, we still refer to the wind directions by their cardinal points. For example, a northern alongshore wind corresponds to a NNW direction (on average).

#### Tidal data

Tidal data were provided by an estuarine model system previously validated for the Tagus estuary [6] (see S1 File). Tidal data were predicted for a specific point located near Cascais (outside the estuary) and situated within the Tagus plume propagation region. The maximum sea surface elevation was 1.81 m, whereas the minimum was -1.77 m (Fig 2C). The maximum daily tidal amplitude attained 3.59 m and the minimum 0.83 m, with a mean range of 2.26 m (Fig 2D).

Tidal influence on river plumes flowing directly into the sea is principally related to high and low tides, but with a less important or even negligible contribution of the spring-neap cycle ([10], in Douro River). However, the fortnightly cycle associated with spring and neap tides of rivers discharging in large estuaries, such as the Tagus, can also modify the plume pattern because of a large water residence time enhancing turbidity [5]. The main differences between spring and neaps are the tidal amplitude and the current velocity, which is more intense during spring tides. During neaps, low velocities are found and residence time within the estuary is changed. In fact, in neaps estuarine outflow is reduced due to low ebb velocities and the water properties are "trapped" inside the estuary, due to low exchange in its mouth. This affects the plume propagation to the coastal ocean as studied by [5]. In a coastal plain estuary like the Tagus, which present a large width in its mixing region (see Fig 1), the estuary remains well mixed due to a large lateral spreading of freshwater through a low depth region. Also, the tide always acts to produce well mixed estuarine conditions. However, different tidal conditions induce changes in current velocities and also in estuarine outflow, and the estuary exports more particulate and solute material during spring than during neaps, as observed by [5]. Therefore, the plume behavior can be different before and after neap or spring tides.

A step forward is given related to the work by [5]. In fact these authors focused on the different propagation and coastal patterns produced by the fortnight cycle and not on the integrated role of the tides and freshwater discharge from the main tributaries. Despite the well mixed characteristics of the Tagus estuary, changes in water properties are found within the estuary, affecting coastal salinity and turbidity [6], which can also affect the region in terms of its biological production (e.g. chlorophyll concentration) [28].

Spring tides were recorded when the daily amplitude was > 75^th^ percentile (2.70 m), whereas neap tides were registered when the amplitude was < 25^th^ percentile (1.83 m). Tides > 0.5 m were regarded as high tide and deemed a low tide when < - 0.5 m while using the predicted sea surface elevation at the moment of the satellite overpass (~ 13:30 UTC for the Aqua satellite).

### Methodology

The effect of each forcing on the Tagus River plume was analyzed through turbidity composites constructed for days fulfilling the conditions, characterizing each driving factor (e.g. discharge > 66^th^ percentile). Turbidity composites were obtained by averaging the valid pixels available for each image. All the situations under scope present an enough number of images to adequately evaluate plume patterns (Table 2). The creation of composites is based on different assumptions.

**Table 2:** Main synoptic conditions analyzed in the study.

Synoptic Situation	Number of days	River Discharge (m^3^s^-1^)			
		Mean	Min	Max	Std
**River Discharge**					
Low	**148**	**45.06**	4.53	76.52	14.38
High	**161**	**374.80**	208.70	942.43	123.98
**Wind condition**					
Northern alongshore	**361**	**374.59**	208.66	991.23	144.08
Southern alongshore	**102**	**439.42**	211.46	995.96	198.63
Eastern cross-shore	**146**	**402.94**	208.10	995.63	173.98
Western cross-shore	**151**	**449.34**	211.41	990.34	181.16
**Fortnightly tidal state**					
Before Spring	**125**	**409.78**	211.99	931.42	169.41
Spring	**178**	**405.53**	208.10	999.01	192.02
Before Neap	**130**	**388.54**	214.29	926.41	162.63
Neap	**212**	**387.27**	208.66	953.30	157.17
**Semidiurnal tidal state**					
High Tide	**237**	**406.23**	197.82	974.16	169.48
Low Tide	**181**	**402.61**	198.29	940.07	171.78

Number of days is referred to the available days representing each condition and used to create the respective composite. The main conditions of Tagus river discharge associated to each situation (mean, minimum, maximum and deviation values) are also shown.

#### NIR vs SWIR_NIR bands for atmospheric correction

In standard MODIS ocean color data processing for atmospheric correction, the ocean is viewed black by the 748 and 869 nm Near-Infrared (NIR) bands. However, some studies indicated the processed NIR ocean color images are not valid for assessing very turbid waters because of the contribution of ocean water-leaving radiance (invalid NIR black ocean assumption) [13, 29]. A combined SWIR_NIR algorithm was developed for very turbid waters since the *black assumption* is generally valid for Short Wave Infrared (SWIR) bands (1240 and 2130 nm wavelenghts). However, the use of SWIR bands introduces greater noise in the processed images [29]. Both atmospheric corrections NIR and SWIR_NIR were evaluated to apply the most suitable one for the moderate turbid areas of the Tagus plume.

**Fig 3:**
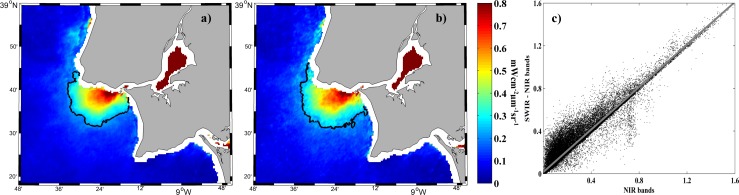
Mean water-leaving radiance (mWcm^-2^μm^-1^sr^-1^) composite at 645 nm for all MODIS images under high river discharge (> 66^th^ percentile) during 2003. Results compare the effect of atmospheric corrections using NIR (a) and SWIR_NIR (b) channels. Scatterplot (c) of corresponding available pixels in each image.

Tagus plume image composites were constructed from the Aqua satellite data for river discharges > 66^th^ percentile occurring in 2003 (Fig 3). Fig 3A shows the atmospheric corrected composite image assembled from NIR bands, whereas Fig 3B displays the composite image created with an alternative SWIR_NIR algorithm described by [29] and used by [8]. Both corrections have been preferably used in turbid plume studies.

Similar composites were obtained by the application of both atmospheric corrections probably due to the moderate local turbid values (Fig 3A and 3B). When valid pixels provided by both corrections were compared, we obtained a linear regression (SWIR_NIR = 0.997 NIR + 0.015), with r^2^ equal to 0.96 (Fig 3C). The linear regression was calculated for the area of plume development. Similar or better linear regressions were achieved (in terms of equivalence between both atmospheric corrections) when we incorporated all river discharges or/and the entire area covered by the images. The comparison allows the application of both correction methods and, although NIR bands can produce significant errors for extremely turbid waters, the moderate turbidity of the Tagus plume mitigates their production.

The normalized standard deviation is an indication of the dispersion (the ratio between the standard deviation and the mean value) associated with both atmospheric correction procedures. It reaches 10.77% for SWIR_NIR and 8.97% for NIR, thus the NIR atmospheric correction is slightly better. SWIR derived products are noisier because SWIR bands were originally designed for atmospheric and land applications and have lower signal-noise ratio (SNR) values [29].

Previous work carried out in the Tagus region [5] and in nearby areas characterized by similar turbidity conditions (ex: the Douro plume [10]; and Ebro plume [11]) made use of the NIR bands for atmospheric correction which is thus applicable to the Tagus plume.

#### Merging data from the Aqua and Terra satellites

Merging daily images of the Tagus plume acquired from both satellites enhances the robustness and precision of the study by increasing the number of available pixels. If a pixel only has a valid value from one satellite, this value is assumed and if it has valid values from both satellites, both values are averaged. This process is made for each pixel in order to obtain a daily image using data from both satellites. Therefore, we must address the differences between both satellites imagery for their suitability in the case of the Tagus plume.

**Fig 4:**
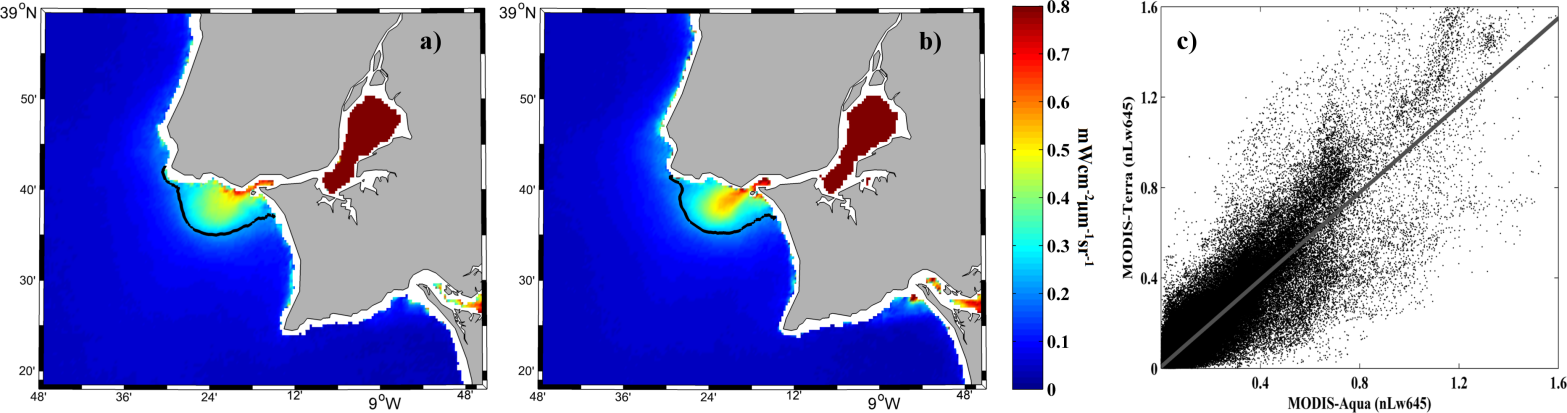
Mean water-leaving radiance (mWcm^-2^μm^-1^sr^-1^) composite at 645 nm for all MODIS images under high river discharge (> 66^th^ percentile) over the period 2003–2015. Results compare data obtained from Aqua (a) and Terra (b) satellites. Scatterplot (c) of corresponding pixels in each image.

Composites created with images from the Aqua and Terra satellites show a similar plume development pattern (Fig 4A and 4B). Composites were generated from 2003 to 2015 during periods of river discharges > 66^th^ percentile. Differences between the composite images are mainly due to the availability of days without cloud coverage for each satellite.

We obtained a good linear regression when the values of daily available pixels acquired from both satellites were compared (MODIS_Terra = 0.964 MODIS_Aqua + 0.007) with a correlation coefficient (r^2^) equal to 0.89 (Fig 4C). The linear regressions had similar or better correlation coefficients when we considered all discharges and/or the entire area depicted in the images.

Our results indicate the differences between the data obtained from both satellites are negligible for the purpose of this research. In summary, merging daily data from both satellites is justified when analyzing the role of river discharge and wind on the Tagus plume development. A difference of a few hours in the overpass of both satellites is negligible to study the mean state of the plume under these drivers. Increasing the percentage of available pixels is especially important for less frequent events such as high river discharges under southern alongshore winds.

However, data merging is not justified when the influence of tides is analyzed, because the tide status is acquired at the instant of the satellite overpass. If data obtained from both satellites were merged, different tidal states (out of phase by 2–3 hours) would be recorded. Thus, the tide influence on plume development was analyzed using imagery from the Aqua satellite to avoid ambiguities in tidal state and because of a greater number of available pixels provided by this satellite.

**Fig 5:**
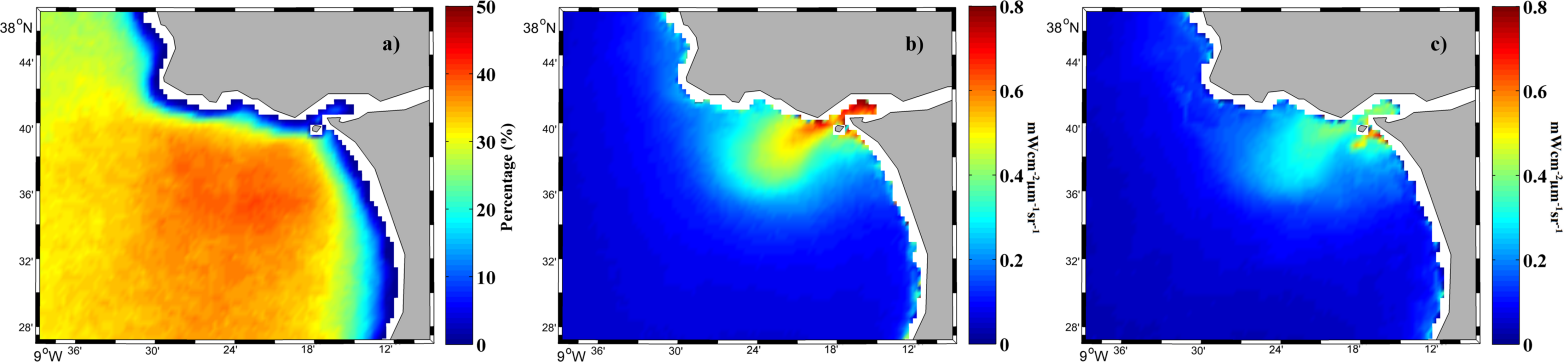
(a) Percentage of valid values, (b) mean value, and (c) standard deviation for each pixel of the composite considering all MODIS images under high river discharge (> 66^th^ percentile) over the period 2003–2015.

Once this analysis was made, it is interesting to know the typical valid data into each pixel of composites, as well as its variance. The percentage of valid values to create a composite when both satellites are merged, was analyzed under high river discharges (above the 66^th^ percentile), when Tagus plume is well developed. Fig 5A shows 40% of valid data in the plume development area, therefore, taking into account the period of study, these pixels include around 650 valid data, which remarks the robustly of the methodology developed. In addition, if mean value of each pixel (Fig 5B) and its deviation (Fig 5C) are compared, a great dispersion in the plume development area is observed, with values around 0.3 mWcm^-2^μm^-1^sr^-1^, a deviation near to mean values (0.5 mWcm^-2^μm^-1^sr^-1^). This behavior is due to the selected percentile, which includes days with discharges ranging between 196 and 997 m^3^s^-1^ and different wind and tidal conditions that influence the turbidity of the area. This implies that pixels in plume development area can reach values between 0.2 (the turbid limit) and above 1.2 mWcm^-2^μm^-1^sr^-1^, showing their high variance.

#### Turbidity threshold

**Fig 6:**
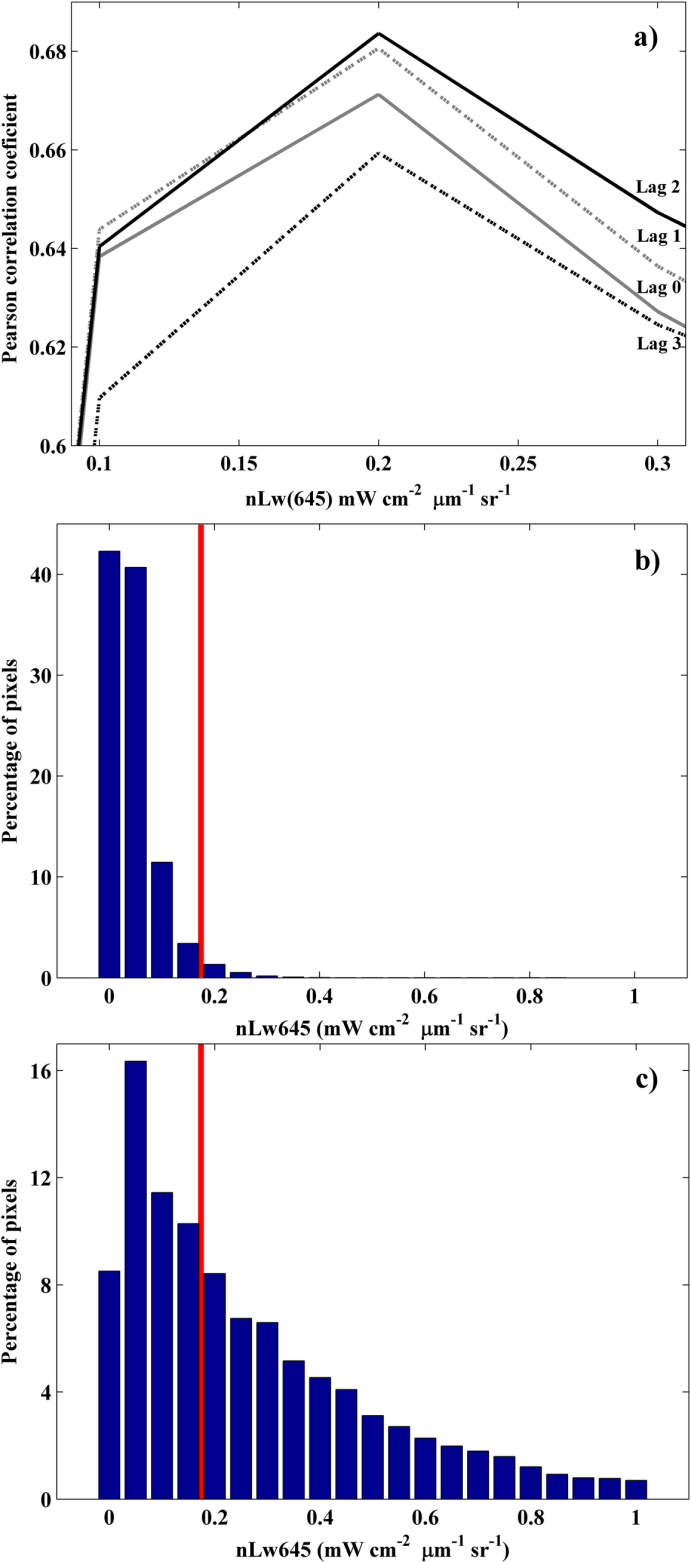
a) Correlation between the daily river discharge and the plume extension for different threshold values. The river discharge is presented for different delays respecting the date of sampling the turbid plume. Only daily images with more than 70% of available pixels covering the area of the sampled plume were utilized. The distribution of pixels for different threshold intervals are shown for images with (b) a negligible plume and (c) a well-developed plume.

A proper definition of the turbid limit is crucial since it allows the distinction of water areas affected by the river plume from the adjacent seawater. We considered two alternative methods which increased the confidence in the obtained threshold. The first method, previously used by [11], evaluates the maximum correlation between river discharge and turbid plume extension under different delays for distinct threshold values (Fig 6A). Only daily images containing > 70% of the available data covering the Tagus plume area were gathered to remove disturbances caused by the lack of valid pixels, resulting in a series containing only 650 valid values. A maximum correlation was obtained for a threshold of 0.2 mWcm^-2^μm^-1^sr^-1^.

The second method, previously used by [8], presents a histogram of the distribution of radiance (nLw645) for days characterized by a negligible plume (Fig 6B) and days showing a well-developed plume (Fig 6C). More than 97% of the pixels were below the 0.2 mWcm^-2^μm^-1^sr^-1^ threshold in situations without plume. Therefore values ranging from 0 to 0.2 mWcm^-2^μm^-1^sr^-1^ can be considered as residual turbidity in the seawater enclosed in the ROFI. In contrast, Fig 6C displays a large percentage of pixels with a value > 0.2 mWcm^-2^μm^-1^sr^-1^ for a well-developed plume.

Both methods confirm the 0.2 mWcm^-2^μm^-1^sr^-1^ threshold as the most appropriate to identify the Tagus turbid plume for a wavelength centered on 645 nm. Although we refer to the Tagus turbid plume, we can consider that the threshold really define the near-field of the turbid plume, which is defined as the oceanic area where the river influence is more intense and the contrast with ocean waters is larger [30, 31]. Thus, pixels above this limit define the plume area where the water differs more from ocean water due to the major influence of material and freshwater discharged by the river, and therefore, where the main processes occur, modifying and differentiating water properties from the adjacent seawater [32]. Although some turbidity can be detected below this value, the material and freshwater are diluted enough to follow a similar dynamic to the ocean water [32].

Therefore, taking into account the definition of the near-field turbid plume, the area surpassing the turbid threshold and where the turbid value of pixels decreases radially from the Tagus mouth was considered as Tagus turbid plume.

#### Dynamical plume parameters

Dynamical classification of plumes by evaluating non-dimensional numbers allows knowing the influence of factors such as outflow inertia, buoyancy forcing and Coriolis effect on plume behavior.

The importance of inertial and rotation processes on plume formation can be evaluated through the Kelvin number at the estuary mouth (*K*_*m*_) [33], such as:

$$K_{m}=\frac{W_{m}}{R_{D}}$$ (1)

where, *W*_*m*_ is the mouth width and *R*_*D*_, the internal Rossby radius, expressed by

$$R_{D}=\frac{\sqrt{g^{\prime }h_{p}}}{f}$$ (2)

where, *f* is the Coriolis parameter, *g’* represents the reduced gravity (*g*′ = *g*(*ρ*_*amb*_–*ρ*_0_)/*ρ*_0_) with *g* as the gravitational acceleration, *ρ*_*amb*_ is the ambient ocean density and *ρ*_0_ the estuarine input density. In addition, *h*_*p*_ represents the plume thickness calculated by the formula used by [2]:

$$h_{p}={\left(2\;L\;v\;h_{0}\frac{f}{g^{\prime }}\right)}^{\frac{1}{2}};$$ (3)

where, *L* is the inflow width, *v* the inflow velocity and *h*_*0*_ the inflow depth.

The Kelvin number can also be calculated using different length scales. The bulk Kelvin number (*K*_*b*_) uses the extension of the plume across-shelf. *W*_*b*_ was obtained from MODIS image composites.

The inflow Rossby number is evaluated to estimate the relative strength of inertial and rotational processes in terms of flow velocity [34], using the equation

$$R_{m}=\frac{u}{fL}$$ (4)

where, *u* is the velocity of the flow current at the estuary mouth.

The densimetric Froude Number (*F*_*m*_) is also determined at the estuary mouth and calculated in the upper water layer to evaluate whether the plume flow is governed by baroclinic or inertial processes [33], thus:

$$F_{m}=\frac{u}{\sqrt{g^{\prime }h_{p}}}$$ (5)

## Results

### River discharge influence on the turbid plume

The correlation coefficients between plume development and river discharge were assessed for different lags in Fig 6A. Tagus plume does not immediately react to river discharge variations despite presenting a high correlation coefficient (above 0.6). The maximum correlation between both variables was obtained with a 2 day delay showing as Tagus plume takes two days, in average, to react to variations in river flow. Therefore, the plume extension is strongly controlled by the discharge occurring for the last three days, including the present day. Attending to this, from now on, the plume extension for *d* (a day), will be compared with average river discharges calculated at *d*, *d*– 1 and *d*– 2.

**Fig 7:**
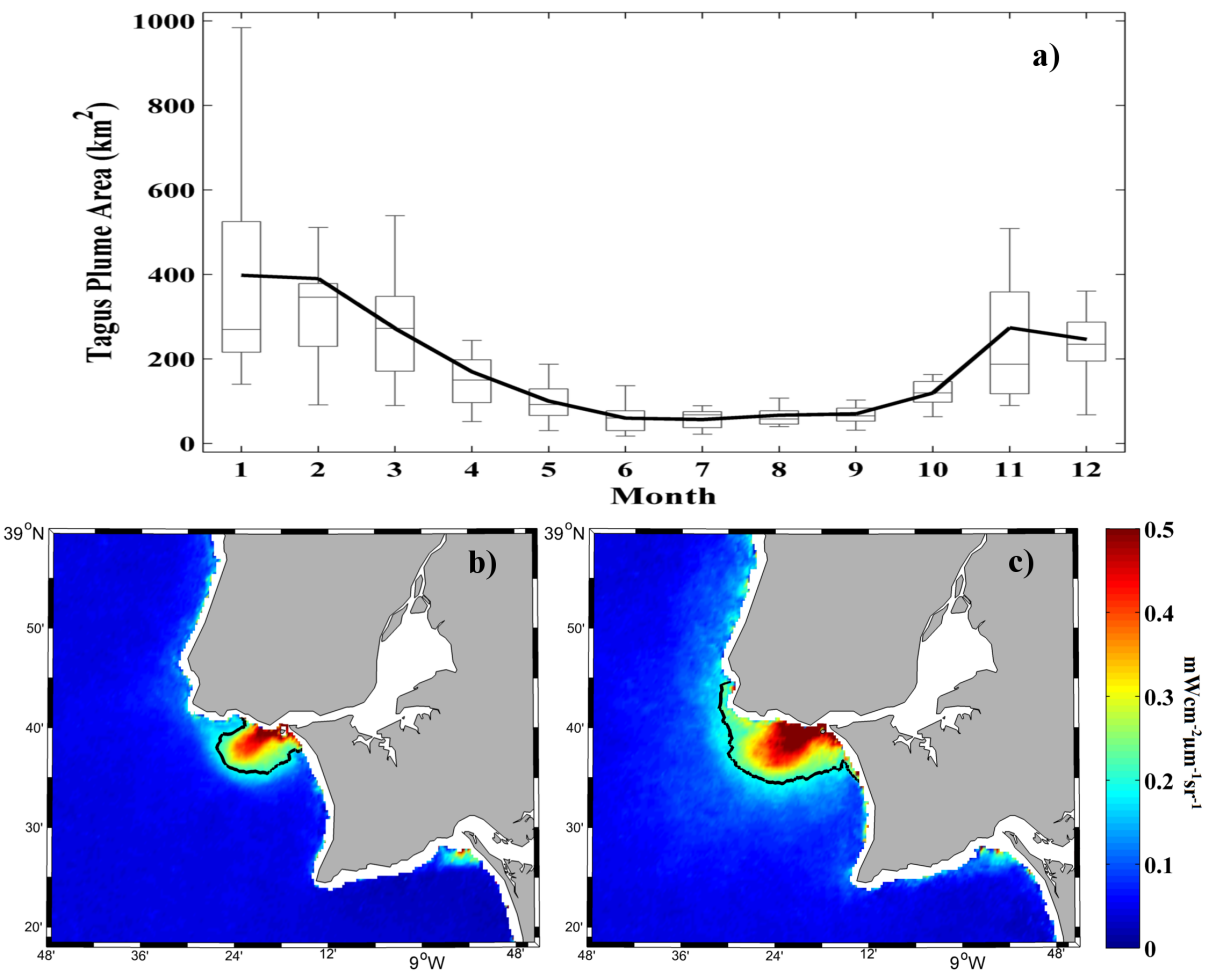
(a) Annual cycle variability of the Tagus River plume extension (km^2^) over the period of 2003 to 2015. Solid black line: monthly average; line inside each box: median; lower and upper whiskers: minimum and maximum, respectively; lower and upper box limits: first and third quartiles, respectively. Mean water-leaving radiance (mWcm^-2^μm^-1^sr^-1^) composite at 645 nm for all MODIS images under (b) low river discharge (< 33^th^ percentile) and (c) high river discharge (> 66^th^ percentile) over the period 2003–2015. The black line represents the turbid threshold (0.2 mWcm^-2^μm^-1^sr^-1^).

Fig 7A presents the annual cycle of the Tagus plume. The observed pattern is consistent to that observed for the river discharge (Fig 2A), with high values of both variables during winter months and low values during summer, which demonstrates the key role of Tagus river discharge on plume development. In quantitative terms, January corresponds to the maximum plume extension (~400 km^2^) and July the minimum (~60 km^2^). Although both present a similar pattern some differences occur that can be attributed to the interaction of winds and tides.

The role of low and high river discharges is also exhibited in Fig 7B and 7C, for image composites of the turbid plume. Only days under calm intervals were selected, that is, days with wind velocities < 2 ms^-1^ lasting for a 2 day period before the date of recording, in order to avoid the wind influence which could mask river discharge impact on river plume. Winds with a northern component are predominant most of the year being favorable to produce superficial upwelling blooms, which can generate radiances not associated with river discharges [5, 35]. When the river discharge is < 33^th^ percentile (< 74 m^3^s^-1^) averaging 44 m^3^s^-1^, the turbid plume is located very close to the river mouth occupying a small area with low turbidity values (Fig 7B). Under high river discharge (> 66^th^ percentile, 198 m^3^s^-1^), with an average value of 396 m^3^s^-1^, the plume extension is much larger, increasing its extension more than double respect to low discharges.

### Wind influence on the turbid plume

The role of the wind was evaluated when the turbid plume area is large enough to be significantly modified by the wind stress. Therefore, we only took the data for the average river discharge > 66^th^ percentile. In addition, days under extreme flows, over the 95^th^ percentile, were avoided in order to remove outliers and maintain similar river discharges (differences lower than 15%) for the different conditions analyzed. This process is necessary in the area under scope because southerly and westerly winds are linked to cyclogenetic processes related to episodes of extreme rainfall associated with high river discharges [36].

**Fig 8:**
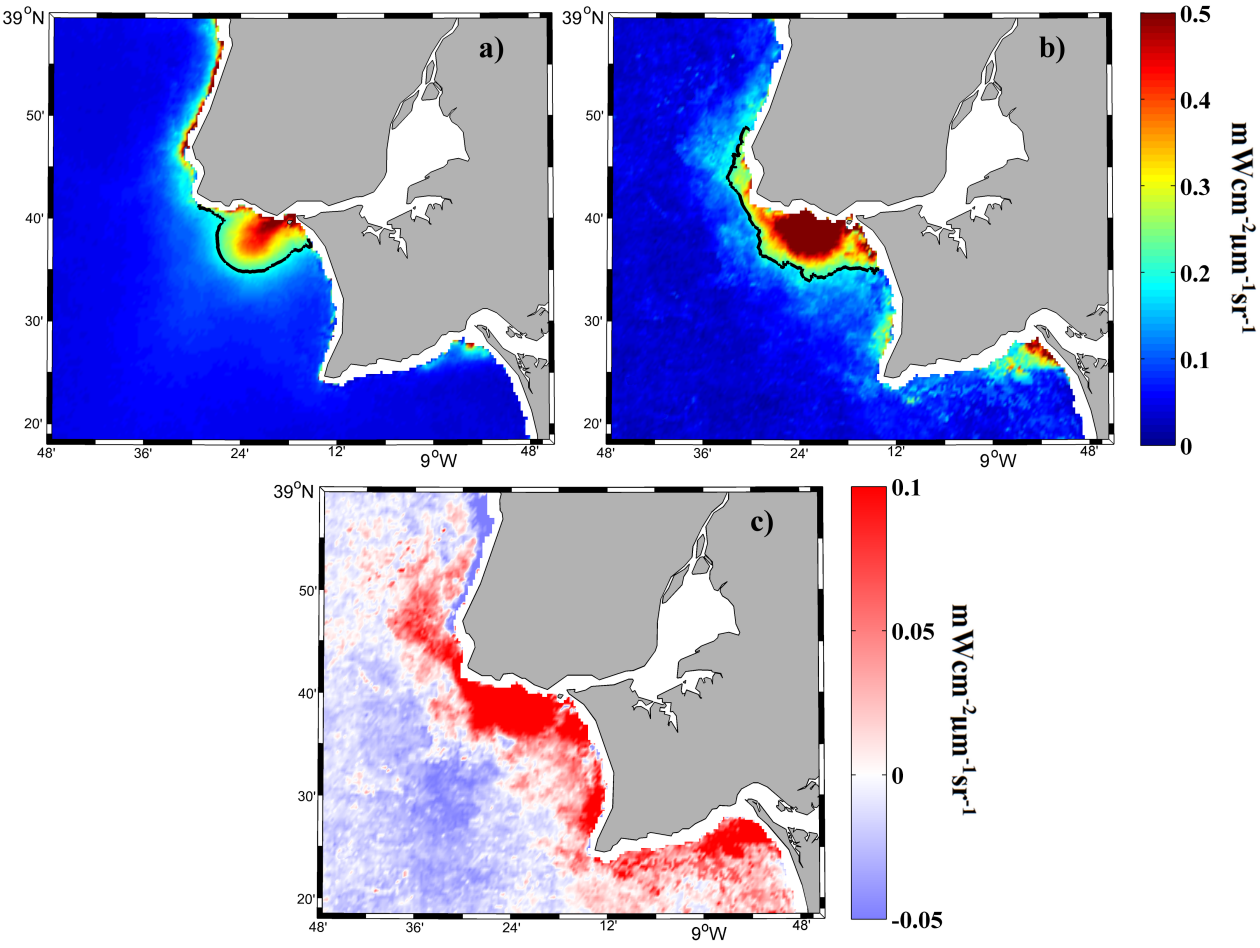
Mean water-leaving radiance (mWcm^-2^μm^-1^sr^-1^) composite at 645 nm for all MODIS images under (a) northern and (b) southern alongshore winds over the period 2003–2015. Black line represents the turbid threshold (0.2 mWcm^-2^μm^-1^sr^-1^). (c) Map of differences on the Tagus turbid plume field under alongshore winds (southern minus northern winds).

Alongshore wind composites are shown in Fig 8. Northern alongshore winds (upwelling) tend to displace the plume material toward the sea due to the offshore Ekman transport. Under these conditions, the velocity of the offshore Ekman current ranges from 0.06 to 0.11 ms^-1^ (at depths of 0 to 5 m) according to [37]. This behavior might be identified in Fig 8A where a rapidly decrease in turbidity values, following a semicircular pattern from the mouth to the ocean, is observed. The dispersion promoted by northern alongshore winds not allow the accumulation of material, which is characterized by the highest turbid values (above 0.5 mWcm^-2^μm^-1^sr^-1^). In addition, a band of high turbidity is observed along the coast north of Cape Roca. This high turbidity band is associated to other processes not related to Tagus discharge since it presents a spatial discontinuity with the Tagus plume. The area characterized by values exceeding the turbid threshold reaches 172 km^2^, with a mean turbid value of 0.36 mWcm^-2^μm^-1^sr^-1^. There, the winds blow with velocities varying from 4 to 8 ms^-1^, with peaks at 12 ms^-1^.

Southern alongshore winds (downwelling) drive the plume against the north coast of the Tagus ROFI, with current velocities ranging from 0.04 to 0.07 ms^-1^ (at depths of 0 to 5 m), due to the onshore Ekman transport. The plume occupies the northern part of the ROFI exceeding it toward the north (Fig 8B). In this case, a semicircular pattern of high turbid values (> 0.5 mWcm^-2^μm^-1^sr^-1^) can be detected around the river mouth. The dispersion and dilution of the material into the ocean is more difficult under these conditions. The turbid plume occupies an area of 306 km^2^ with a mean turbidity value of 0.43 mWcm^-2^μm^-1^sr^-1^. The southern alongshore wind velocities vary from 4 to 8 ms^-1^, with peaks of 12 ms^-1^.

**Fig 9:**
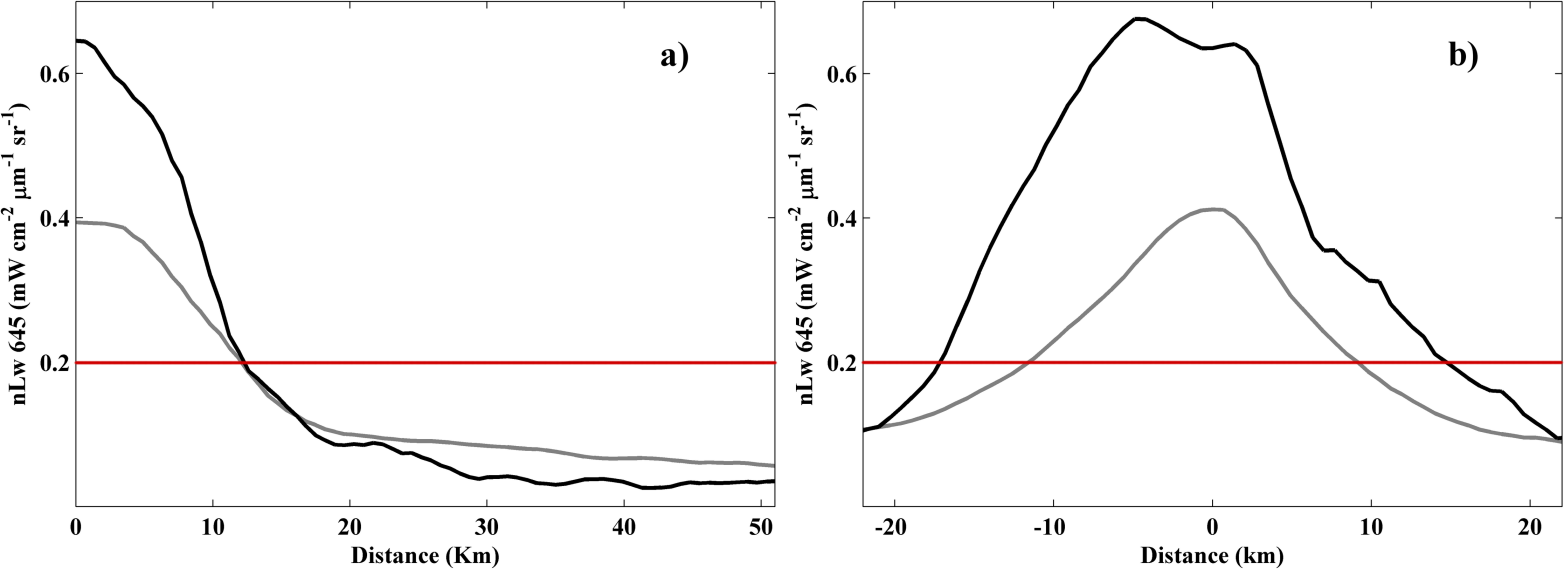
Transect of turbidity for alongshore winds for the (a) perpendicular and (b) parallel directions refer to ROFI coast (see Fig 1). Black line represents southern alongshore winds and grey line northern alongshore winds. Red line represents the turbid threshold. The zero marks the river mouth and negative distances refer to the region located northern to the river mouth.

Turbidity along a transect perpendicular to ROFI coast allows us to better know the effects provoked by both types of alongshore winds in terms of plume dispersion (Fig 9A). Under upwelling winds maximum turbidity values are around 0.4 mWcm^-2^μm^-1^sr^-1^ near the mouth (grey line, Fig 9A), descending moderately and maintaining detectable values large distances over the ocean (~ 50 km), suggesting the dispersion and dilution of material seaward. On the other hand, turbidity values surpass 0.6 mWcm^-2^μm^-1^sr^-1^ near coast under downwelling winds (black line, Fig 9A), diminishing drastically until reach negligible values from 30 km. This shows as the majority of the material exported by the river is retained near coast, probably due to the reduction of the cross-shore transport. In addition another transect starting at the Tagus mouth and extending along the north ROFI coast (negative values) and along the south ROFI coast (positive values) was evaluated to know the extension of the near-field turbid plume under alongshore winds (Fig 9B). Downwelling winds promote higher turbid values than upwelling winds, maintaining values above 0.2 over a larger extension on both sides of the mouth, and especially to the north ROFI coast (black line, Fig 9B). Upwelling winds show a similar pattern on both sides of the mouth with values descending rapidly below the turbid limit (grey line, Fig 9B), which can be attributed to the dispersion promoted. Therefore, although material exported by the river can reach greater distances over the ocean under upwelling winds, this also cause a strong dispersion into ocean water. It is suggested the plume material dispersion and dilution into ocean water makes turbidity values rapidly reach values lower than the turbid limit, reducing the area occupied by the near-field plume. On the other hand, downwelling winds maintain higher values of turbidity near coast since limit its dispersion on seawater, provoking a bigger near-field plume.

**Fig 10:**
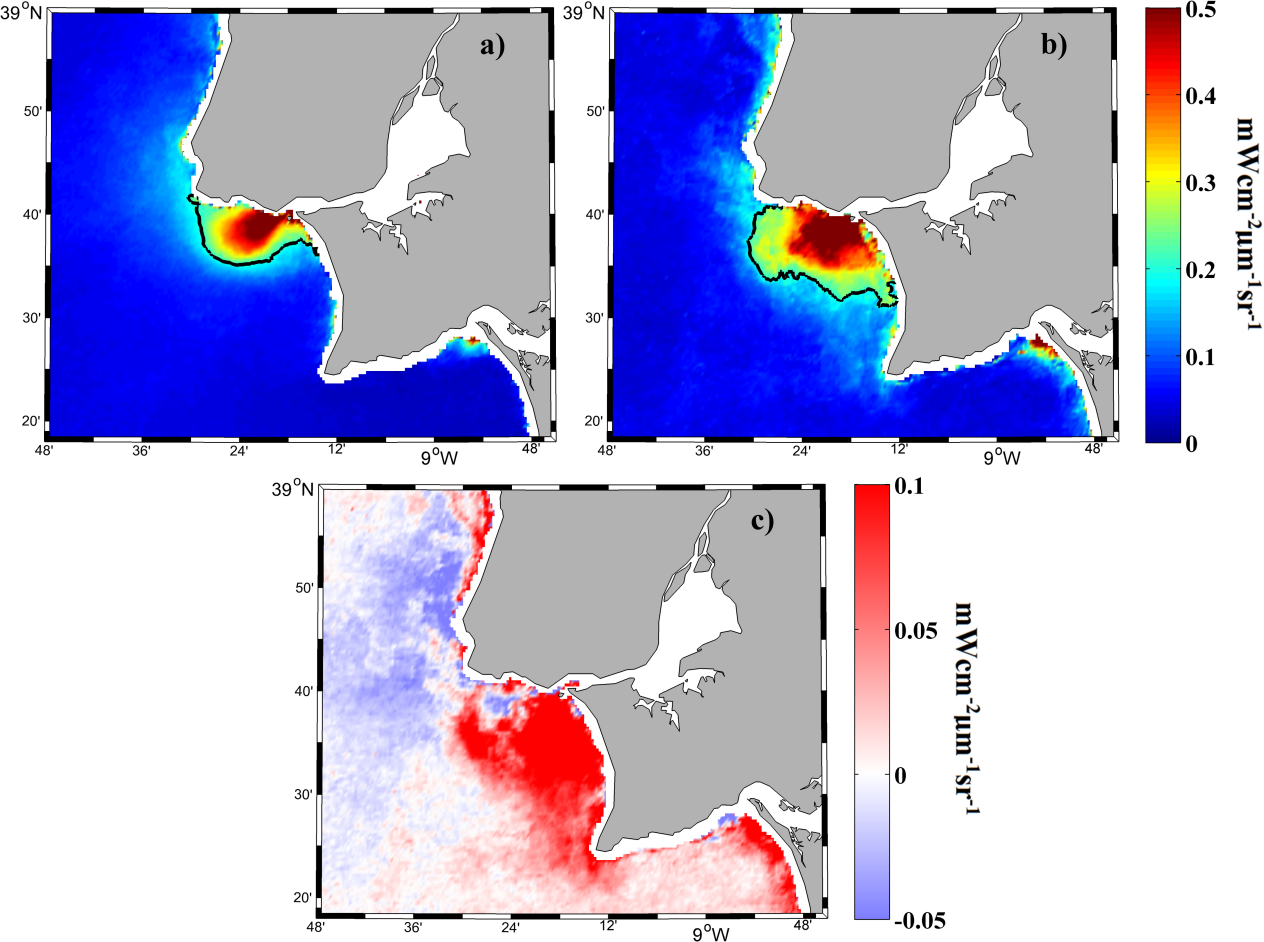
Mean water-leaving radiance (mWcm^-2^μm^-1^sr^-1^) composite at 645 nm for all MODIS images under (a) eastern and (b) western cross-shore winds over the period 2003–2015. The black line represents the turbid threshold (0.2 mWcm^-2^μm^-1^sr^-1^). (c) Map of differences on the Tagus turbid plume field under cross-shore winds (western minus eastern winds).

The effect of cross-shore winds on the Tagus plume is shown in Fig 10. Eastern cross-shore winds (seaward) will stretch the plume offshore, promoting the dispersion of the material on the adjacent seawater. Therefore, the highest turbid values, exceeding 0.5 mWcm^-2^μm^-1^sr^-1^, are only detected in the area nearest to river mouth (Fig 10A). The turbid plume is located in the northern part of the ROFI occupying an area of 187 km^2^ with an average turbid value of 0.39 mWcm^-2^μm^-1^sr^-1^. Eastern cross-shore winds blow with velocities ranging from 2 to 6 ms^-1^, with peaks of 12 ms^-1^.

Western cross-shore winds (landward) retain Tagus plume material inside the ROFI in both alongshore directions, reducing its spreading in ocean waters. Therefore these winds are capable to maintain high turbid values along a greater area, as shows Fig 10B, occupying much of the Tagus ROFI filling a total extent of 389 km^2^ with a mean value of 0.41 mWcm^-2^μm^-1^sr^-1^. The retention of plume material toward the coast produces a visible pattern around the river mouth, characterized by high turbid values (> 0.5 mWcm^-2^μm^-1^sr^-1^), although less extent than under southern alongshore winds. Western cross-shore wind velocities range from 4 to 8 ms^-1^, with peaks of 14 ms^-1^.

**Fig 11:**
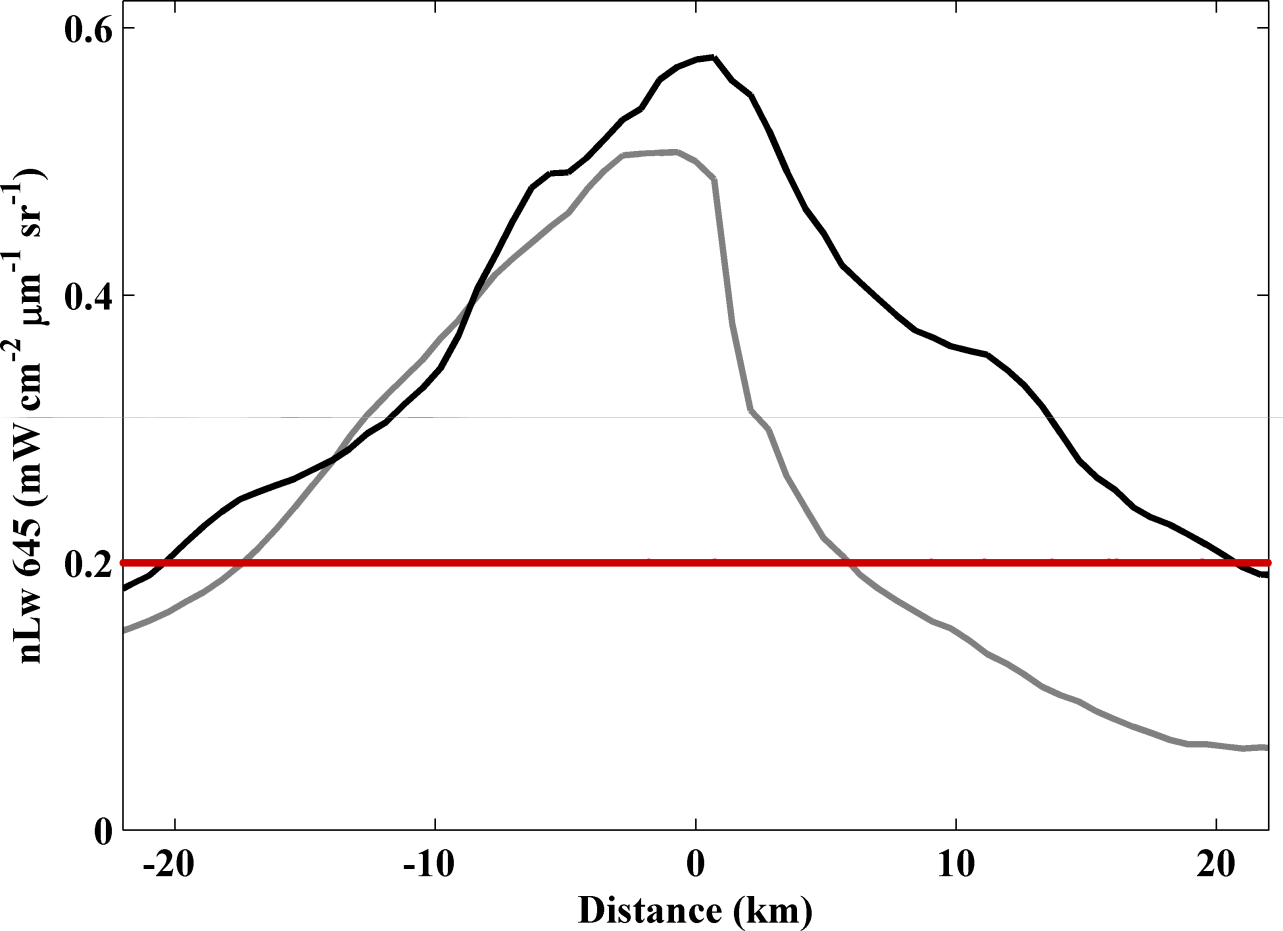
Transect of turbidity for cross-shore winds for the parallel direction refer to ROFI coast (see Fig 1). Black line represents western cross-shore winds and grey line eastern cross-shore winds. Red line represents the turbid threshold. The zero marks the river mouth and negative distances refer to the region located northern to the river mouth.

A transect was also considered to analyze the plume movement under each type of cross-shore wind (Fig 11). In this case, as these winds tend to move the plume along the north coast of the ROFI (seaward winds) or to both sides of the mouth (landward winds), the transect runs from the mouth to both alongshore directions being positive in the southward direction. Under landward winds a maximum turbidity near to 0.6 mWcm^-2^μm^-1^sr^-1^ is detected near the mouth decreasing in a similar way to both sides of the mouth (black line, Fig 11). On the other hand, this pattern is only observed to the north side of the river mouth under seaward winds (grey line, Fig 11). In this case, turbidity reach a maximum value of 0.5 mWcm^-2^μm^-1^sr^-1^ at the river mouth diminishing abruptly southward. Results show as landward winds retain plume material in both alongshore directions with a near-field plume reaching larger distances whereas seaward winds move the material only along the north coast of the ROFI avoiding the formation of high values of turbidity due to the offshore movement promoted.

The principal characteristics of the Tagus plume are summarized in Table 3 under different meteorological synoptic conditions. Overall, the near- field turbid plume reaches its highest extension when western cross-shore winds prevail.

**Table 3:** Characteristics and implications of the Tagus turbid plume under wind forcing.

Meteorological synoptic conditions		Plume characteristics		Implications
River (m^3^s^-1^)	Wind (calm discarded)	Extent (km^2^)	Mean turbid value (mWcm^-2^ μm^-1^ sr^-1^)	
375	Northern alongshore	172	0.36	Intense offshore transport
439	Southern alongshore	306	0.43	Retention north of ROFI
403	Eastern cross-shore	187	0.39	Offshore and northward dispersion
449	Western cross-shore	389	0.41	Retention occupying the whole ROFI area

### Tidal influence on the turbid plume

**Fig 12:**
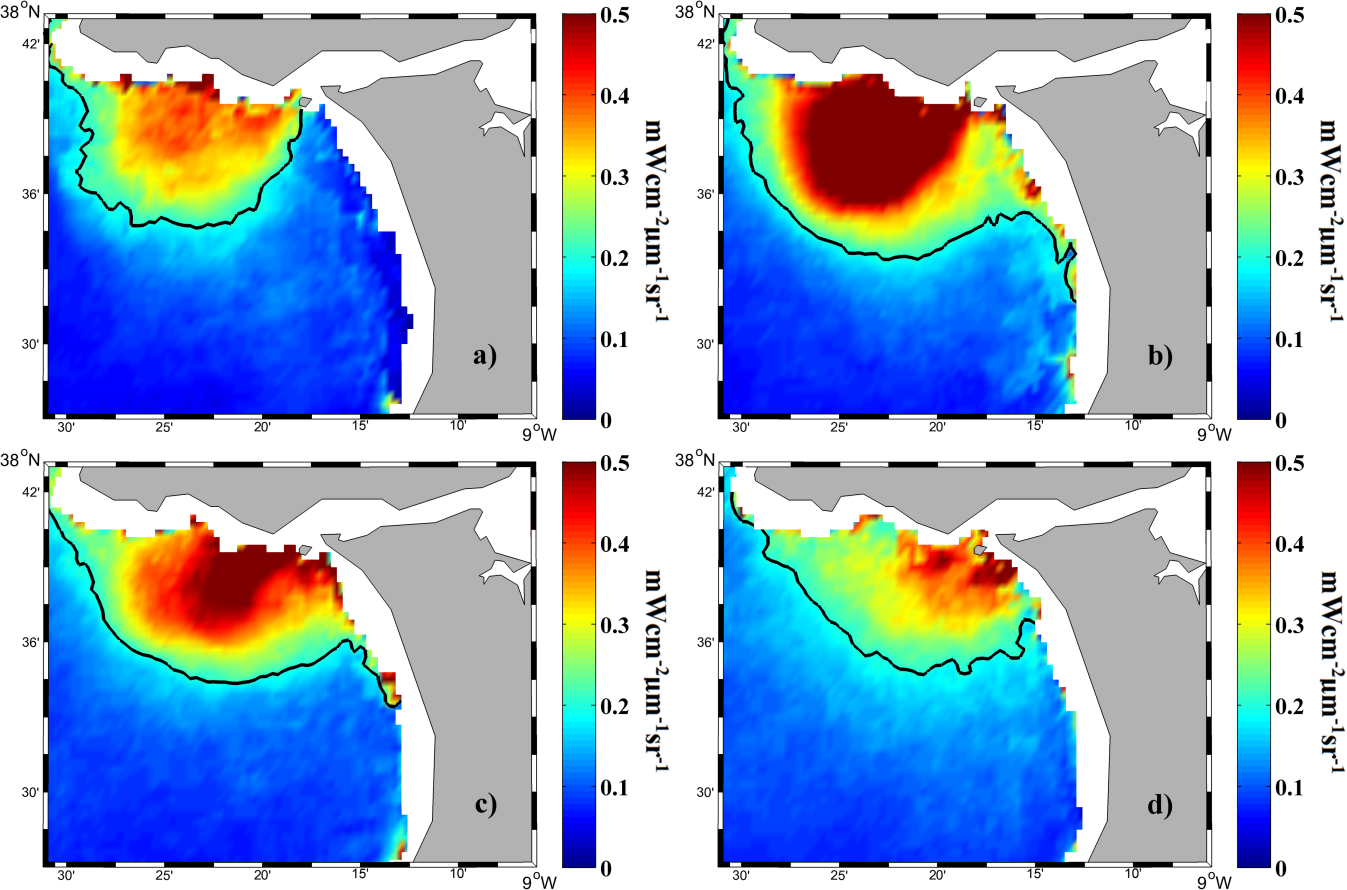
Mean water-leaving radiance (mWcm^-2^μm^-1^sr^-1^) composite at 645 nm for all MODIS images (a) before spring tides, (b) during and after spring tides, (c) before neap tides and (d) during and after neap tides, over the period 2003–2015. The black line represents the turbid threshold (0.2 mWcm^-2^μm^-1^sr^-1^).

The fortnightly (spring and neap) and semi-diurnal (high and low) tidal periodicities were analyzed using imaging composites of turbidity obtained during days showing a river discharge > 66^th^ percentile and avoiding extreme flows above 95^th^ percentile for the reasons commented in the previous section. In addition, the tidal composites can be affected by the upwelling blooms out of ROFI limits, causing radiance not associated to river discharge, as it was commented in section 3.1. Therefore, as Tagus plume under the conditions described not surpass the Tagus ROFI, images were reduced to this area in order to analyze tidal influence in detail, taking into account that tidal effect on plumes is noticeable in the area close to the estuary mouth [10]. For this purpose, we composed average nLw645 fields before spring and neap tides (Fig 12A and 12C) and during-after spring and neap tides (Fig 12B and 12D) to evaluate the influence of the fortnightly tidal periodicity. During-after spring tides maximum turbid values and plume extension were obtained (0.43 mWcm^-2^μm^-1^sr^-1^ and 302 km^2^, respectively). In fact, it is the only case where a distinguishable circular pattern of high turbid values (> 0.5 mWcm^-2^μm^-1^sr^-1^) is detected around the river mouth. The minimum values were obtained during-after neap tides (0.31 mWcm^-2^μm^-1^sr^-1^ and 141 km^2^, respectively), where turbid values do not surpass 0.4 mWcm^-2^μm^-1^sr^-1^. The mean turbid value and extension of the Tagus plume indicate an intermediate status before spring and neap tides, although values are larger before neap tides.

**Fig 13:**
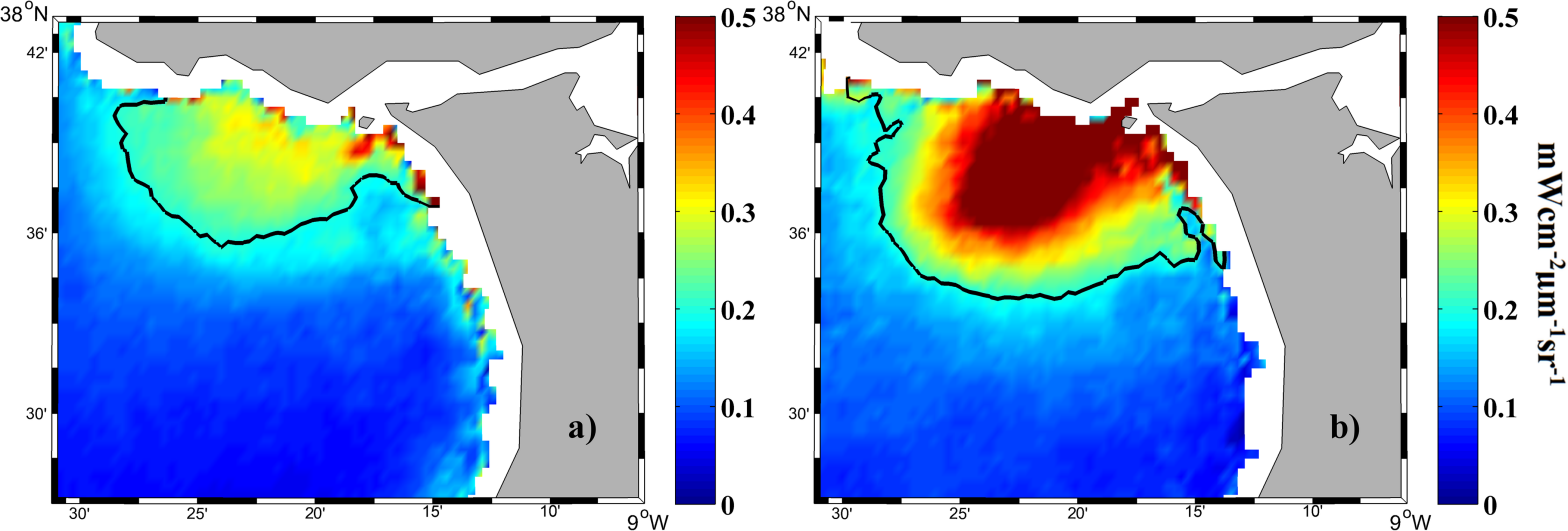
Mean water-leaving radiance (mWcm^-2^μm^-1^sr^-1^) composite at 645 nm for all MODIS images for (a, b) high and low tides during-after spring tides and (c, d) high and low tides during-after neap tides, over the period 2003–2015. The black line represents the turbid threshold (0.2 mWcm^-2^μm^-1^sr^-1^).

High and low phases of tide were evaluated in the middle of the fortnightly cycle, out of spring or neap tides, in order to isolate the semi-diurnal effect (Fig 13). The results reveal the plume extension to be larger and the mean turbid value higher under low tides. In addition, pixels above 0.5 mWcm^-2^μm^-1^sr^-1^ are only detected under low tides whereas values are below 0.4 mWcm^-2^μm^-1^sr^-1^ under high tides. In quantitative terms, Tagus plume reaches an extension and mean turbidity of 188 km^2^ and 0.29 mWcm^-2^μm^-1^sr^-1^, respectively for high tides, and 294 km^2^ and 0.39 mWcm^-2^μm^-1^sr^-1^, respectively under low tides.

The characteristics of the Tagus plume under tidal synoptic conditions are summarized in Table 4. Both semi-diurnal and fortnightly periodicities have an important impact on the size and turbidity of the Tagus River plume. Here, the highest differences in river discharge are around 10%, smaller than previously mentioned when analyzing the effect of wind. Thus, tidal analysis is not conditioned by variations in the average river discharge.

**Table 4:** Characteristics and implications of the Tagus turbid plume under tidal forcing.

Tidal synoptic conditions		Plume characteristics		Implications
River (m^3^s^-1^)	Tidal state	Extent (km^2^)	Mean turbid value (mWcm^-2^ μm^-1^ sr^-1^)	Fortnightly periodicity
410	Before spring	198	0.33	Intermediate situation
406	During/after spring	302	0.43	Largest plume extension with intense turbidity
389	Before neap	242	0.38	Intermediate situation
387	During/after neap	141	0.31	Smallest plume extension with low turbidity
406	High	188	0.29	Smaller plume extension with less intense turbidity
403	Low	294	0.39	Larger plume extension with more intense turbidity

### Plume parameters

The Tagus plume dynamic was evaluated under river discharges > 66^th^ percentile, which define the scope of this study. The flow velocity at the estuary mouth usually varies between 0.4 ms^-1^ at neap tides and 1.4 ms^-1^ at spring tides [16].

The Kelvin number at the Tagus estuary mouth (K_m_) is < 1 under both flow conditions (Table 5). This suggests that the Tagus plume is mainly controlled by inertial processes at the estuary mouth, with the river discharge likely forming a re-circulating bulge at the front [33]. This hypothesis is corroborated by a Rossby number (R_m_) > 1 under all typical flow conditions. The Rossby number indicates the great influence of inertial processes and a negligible impact of rotational ones at the estuary mouth. The Froude number (F_m_), which is < 1 under low flows, reveals a subcritical flow and the importance of the stratification process, with the plume tending to form a far-field region upon river discharge from the estuary. At medium-to-high flow, the Froude number is > 1. This implies a supercritical flow dominated by inertial processes. The supercritical values also demonstrate the plume ability to generate a near-field region which is a mixing area characteristic of prototypal plumes [34].

**Table 5:** Dynamical parameters of the Tagus plume.

	*low current* *(0*.*4 ms*^*-1*^*)*	*intermediate current* *(0*.*9 ms*^*-1*^*)*	*high current* *(1*.*4 ms*^*-1*^*)*
*h*_*p*_ *(plume thickness)*	2.49 m	4.18 m	6.16 m
*R*_*D*_ *(Radius Deformation)*	6531 m	8462 m	10273 m
*W*_*b*_ *(Plume extent)*	6195 m	17699 m	23008 m
***Bulk Kelvin number (K***_***b***_***)***	**0.93**	**2.06**	**2.21**
***Mouth Kelvin number (K_m)_***	**0.31**	**0.24**	**0.19**
***Mouth Rossby number (R*_m_)**	**2.21**	**4.98**	**7.74**
***Mouth Froude number (F*_m_)**	**0.68**	**1.18**	**1.51**

At low flow, the bulk Kelvin number (K_b_) is slightly smaller than 1, therefore the Tagus plume is controlled by inertial processes although rotational ones are also important. When the flow is medium-to-high, K_b_ is > 1. This defines the plume as “Large-scale”, indicating the important role of the Coriolis effect when the Tagus plume is developed [33] (Table 5).

## Discussion

The analysis of the Tagus turbid plume under its main forcing shows a marked control of river discharge on plume development. This is clearly observed when both annual patterns are compared (Fig 7A), showing a similar behavior with high values in winter months and low ones during summer. Therefore, Tagus plume extension increases with river discharge, as can be observed in Fig 7B and 7C where Tagus plume occupies more than double area under high discharges than under low ones. In addition, a semicircular pattern formed by the highest turbid values (> 0.5 mWcm^-2^μm^-1^sr^-1^) is clearly observed when Tagus discharge is high while it is very unlike to find under low discharge. The observed findings are attributed to the greater amount of material exported from the estuary as the flow increases [4], since the flow increasing enhances the capability of the river to drag suspended matter to the mouth [11]. This behavior was previously observed in other rivers along the Iberian Peninsula coast for which the river discharge is the main support to plume development [10, 11]. However, although Tagus turbid plume is highly dominated by the discharge pattern, it is not able to adapt immediately to its variations, taking 2 days to cope with flow fluctuations, in contrast with other Iberian rivers, which respond immediately to discharge variations (e.g. the Douro River, [10] and the Ebro River, [11]). This delay is probably due to the long residence time of water caused by the shape and size of the estuary. The Tagus River debouches into a great estuary before reaching the ocean while the other Iberian rivers flow directly into the sea. The observed delay could be in part attributed to the distance of the discharge gauge to the estuary mouth. To check this, the river discharge data from the Almourol station located ~ 90 km from the estuary entrance and from the Ómnias station situated only 45 km from the estuary entrance are compared for a period lasting from 1990 to 2002; the Ómnias station stopped providing data in 2002. Results show the Almourol and Ómnias stations river discharge are in phase. This confirms that the observed delay between plume development and river discharge is related to the shape and characteristics of the Tagus estuary, which is constituted of a large region of mixing water, approximately 25 km long x 10 km wide, extending from the mouth to the upper reaches of the estuary.

Once Tagus plume is well developed (under high discharges) the wind impact on its dispersion is of great importance. Upwelling favorable winds transport plume material large distances over the ocean due to the offshore Ekman transport, promoting its dispersion and dilution in ocean water (Fig 9A). This movement may be detected in Fig 8A as a rapidly decrease in the turbidity, which provokes that very high turbid values (> 0.5 mWcm^-2^μm^-1^sr^-1^) were not detected near coast. This behavior is in agreement with the reduction of salinity differences between coastal waters affected by river discharge and ocean waters due to the mixing promoted by upwelling winds shown in previous works [38]. The difference in the turbid plume field between alongshore winds (downwelling minus upwelling winds) represented in Fig 8C shows a dominance of negative values (dominance of upwelling winds) in the far field in front of the river mouth. This fact suggests the capability of upwelling winds to transport plume material affecting large distances over the ocean, which was previously found for Tagus plume through numerical models [4] and agrees with previous works that detected the offshore advection of plumes caused by upwelling winds [38, 39]. This dispersion of plume material provokes that although some turbidity can be detected to great distances over the ocean, its value is rapidly below the turbid limit, and therefore, the area occupied by turbidity values surpassing the limit (near-field plume) is smaller. This can contrast with the studies mentioned above which described a larger plume promoted by upwelling winds due to the greater expansion over the ocean. These studies account the entire area where salinity is lower than oceanic one due to discharge influence, whereas in our case we are centered in the near-field turbid plume, the area where the contrast is greater. From this point of view the smaller area is obtained under winds that tend to extend the plume material over the ocean because promote a lower contrast with ocean water, and therefore, the area characterized by a strong contrast with ocean water is reduced. In addition, a great bulge was not clearly observed in front of Tagus river mouth under upwelling winds as it was observed in other Iberian rivers [10] probably due to the shape of Tagus coast, which limits its formation.

A coastal turbid fringe is also detected north of cape Roca (Fig 8A and 8C) under upwelling winds. This fringe of turbidity is not caused by the material exported by Tagus River neither by the material discharged by rivers located northward, because there are not main rivers in that area. The cause supporting the appearance of this fringe of turbidity north of Tagus ROFI under upwelling winds was explained by [5, 35]. They found that this type of winds are able to promote upwelling blooms causing radiance not associated with the river discharge. In fact, [5] found that under extreme high discharges, Tagus plume can be large enough to reach these upwelling blooms making both patterns undistinguishable. However, with our methodology, this fringe of turbidity is not spatially continuous to the Tagus plume, and therefore, we can delimit the near-field plume and differentiate it from the turbid fringe caused by upwelling blooms.

Downwelling winds dominate the area near coast in the north of Tagus ROFI due to the onshore Ekman transport promoted, retaining the material exported by the river in that area and limiting its dilution in the ocean water (Figs 8B and 9A). This might explain the high turbid values detected in Fig 8B and promotes that values surpassing the turbid limit occupy a larger area (Fig 9B). This behavior is corroborated by Fig 8C where a fringe of positive values (dominance of downwelling winds) dominate the near shore area, extending northward to Tagus ROFI. This behavior is in a good agreement with previous works that shows as downwelling winds dominate the coastal area on the side of the mouth toward wind blows [10, 39, 40, 41]. However, these studies detected a fringe of plume material along the coast following wind direction which was not observed northward of cape Roca. This is probably caused by the retention of Tagus plume against north of ROFI due to the shape of the bay, limiting that plume surpasses this area, which prevents its propagation northward. In the case of studies mentioned above there are no geological barriers avoiding the formation of this coastal band following wind direction. This fact and the time delay between river discharge and river plume variations caused by the size and the shape of the Tagus estuary highlights the importance of coastal geomorphology in the Tagus case.

Eastern cross-shore winds tend to push the plume material offshore, which causes that the highest values of turbidity (> 0.5 mWcm^-2^μm^-1^sr^-1^) were scarcely detected not allowing the material accumulation near coast (Figs 10A and 11). The near-field turbid plume occupies a smaller area located north of ROFI. Unlike the upwelling winds that move the material in the perpendicular direction to the ROFI coast, seaward winds move the material predominantly offshore following the north coast of the ROFI and toward the north when the ROFI is surpassed, following the coastline geometry and probably affected by Coriolis deflection to the right in the Northern Hemisphere. This behavior is clearly observed when the map of differences between cross-shore winds (landward minus seaward winds) is assessed (Fig 10C). The negative values (dominance of seaward winds) are observed to the north of river mouth showing as eastern winds tend to displace the plume offshore and to the north when the ROFI is surpassed. The offshore movement of the plume under eastern winds and the deflection to the right is also observed in Douro plume [10]. On the other hand, landward winds retain most of plume material against the coast in both alongshore directions (Fig 11), limiting its spreading in ocean water and therefore, maintaining values above the limit in a greater area inside the ROFI (Fig 10B). In fact, the highest turbid values form a defined semicircular pattern around the mouth. This behavior is corroborated by Fig 10C where positive values (dominance of landward winds) dominate the area enclosed by the bay excepting the north part, which is also affected by seaward winds.

Then, plume material is affected in a different manner depending on the predominant wind direction. Upwelling winds tend to disperse plume material over the ocean following a perpendicular direction to the ROFI coast, provoking a near-field plume which occupies the smallest area located in the north of ROFI and defined by the lowest mean turbid value (Figs 8A, 8C and 9, Table 3). Downwelling winds provoke that Tagus plume occupies all the north of ROFI since maintain the material discharged by the river in that area due to the onshore Ekman transport generated, which limits the transport of material to other areas. This promotes a near-field plume defined by the highest mean turbid value and with an inclination to the north (Figs 8B, 8C and 9, Table 3). Seaward winds displace plume material toward the ocean and following the north coast when the ROFI is surpassed, causing a small near-field plume characterized by low turbidity values preserved in the north of ROFI (Figs 10A, 10C and 11, Table 3). Finally, landward winds retain plume material in both alongshore directions, which provoke the greatest near-field plume characterized by high turbid values, which occupies a large part of the area enclosed by the bay due to the inhibition of cross-shore transport (Figs 10B, 10C and 11, Table 3).

Tagus dynamic is also affected by fortnightly and semidiurnal tidal cycles. The effect of overlapping both cycles is not observed in other main rivers analyzed in the Iberian Peninsula. Ebro plume is not affected by tides due to the micro-tidal regime of Mediterranean Sea [42], and Douro plume is only affected by the semidiurnal cycle, although it has the same mesotidal Atlantic regime than Tagus. This fact is due to Douro River directly flows into the sea, whereas Tagus presents a great estuary before debouching into the ocean. Large estuaries, as Tagus, generate a longer water residence, which can modify turbidity patterns according to fortnightly tidal cycle [43] whereas the residence time in the Douro estuary is too short to observe this effect [10].

A clear pattern of variability according to the fortnightly period is observed in the Tagus plume (Fig 12). Tagus near- field plume reaches the maximum extension and turbidity during spring tides descending to its minimum values during neap tides, with an intermediate plume observed before neap tides (Table 4). The same behavior occurs between neap and spring tides. The extension of the near- field and plume turbidity increase to reach maximum values during spring tides, and therefore, an intermediate plume is observed before spring tides (Table 4). The observed patterns can be attributed to a larger turbid plume ejected from the estuary during and after spring tides, enhanced by the ebb-dominated Tagus estuary [20]. These results are in good agreement with those of [5, 6].

The semidiurnal tidal cycle also presents an important impact on Tagus plume development. In fact, a greater and more turbid plume is observed under low tides in comparison with the situation under high tides (Fig 13 and Table 4). This behavior is attributed to the plume retraction provoked by the high tide, which limits its development, as was previously observed in other plumes on the Western Iberian coast [10].

## Conclusions

The spatial and temporal variability of the Tagus plume was evaluated considering the effects of river discharge, wind and tide from 2003–2015. The study was carried out using composites constructed of daily images taken from the MODIS Aqua and Terra satellites. The study allowed us to reach the following conclusions:

- The maximum correlation between river discharge and the Tagus plume extension is obtained with a lag of 2 days, i.e., plume at day *d* is compared with the runoff at day *d-2*. The plume does not react immediately to changes in river discharge due to the geomorphology and dimension of the estuary.
- The Tagus plume occupies a small area located near the estuary mouth under low river discharge. The plume shows an increase in extension and turbidity at higher river discharges, showing plume dependence on river flow.
- The influence of wind was investigated under high river discharges. Maximum near- field plume extension (389 km^2^) was observed under western cross-shore winds whereas the maximum mean turbidity (0.43 mWcm^-2^μm^-1^sr^-1^) was induced by southern alongshore winds. Both wind conditions limit the dispersion of material exported by the river in ocean waters, which maintains values above the turbid limit over a large area. On the other hand, northern alongshore winds produced the minimum near- field plume extension and turbidity (172 km^2^ and 0.36 mWcm^-2^μm^-1^sr^-1^, respectively). Eastern cross-shore winds also cause low plume extension and turbidity probably because both wind conditions favor the material dispersion in ocean waters provoking a rapidly decrease in turbidity values and therefore, in the area occupied by the values surpassing the turbid limit.
- The role of tides was analyzed under high river discharges. Low tides create larger near- field turbid plumes with higher turbidity values than at high tides. In addition, the near- field turbid plume reached its maximum extension (302 km^2^) and mean turbidity (0.43 mWcm^-2^μm^-1^sr^-1^) during and after spring tides, when we consider the fortnightly tidal cycle. The minimum near- field plume extension and mean turbid value are obtained during and after neap tides (141 km^2^ and 0.31 mWcm^-2^μm^-1^sr^-1^, respectively). Therefore, semi-diurnal and fortnightly tide periods, are important for the plume size and turbidity. When the two periods overlap, the maximum near- field plume extension and mean turbidity were acquired at during-after spring tides under low tides, whereas the minimum near- field plume and mean turbidity were observed during-after neap tides under high tides.

## Supporting information

S1 File Tidal constituents list used to reconstruct the tidal series. (DOCX)
