# Supplementary material for: Influence of main forcing affecting the Tagus turbid plume under high river discharges using MODIS imagery
Source: PLoS One. 2017 Oct 26;12(10):e0187036. doi: 10.1371/journal.pone.0187036 (PMC5658172; doi:10.1371/journal.pone.0187036)
Supplement: S1 File — (DOCX) [file pone.0187036.s001.docx]

Supporting Information for

**Influence of main forcing affecting the Tagus turbid plume under high river discharges using MODIS imagery**

**D. Fernández-Nóvoa^1*^, M. Gómez-Gesteira^1^, R. Mendes^2^, M. deCastro^1^, N. Vaz^2^, J.M. Dias^2^**

^1^ EPHYSLAB, Environmental PHYsics LABoratory, Facultad de Ciencias, Universidad de Vigo, Ourense, Spain.

^2^ CESAM, Physics Department, University of Aveiro, Aveiro 3810-193, Portugal

**Contents of this file:** Tidal constituents list used to reconstruct the tidal series (from Vaz et al., 2011).

Tidal amplitude and phase with 95% CI estimates

tide freq amp amp_err pha pha_err snr

SA 0.0001141 0.0001 0.000 86.67 75.30 0.72

SSA 0.0002282 0.0001 0.000 199.40 72.00 0.75

MSM 0.0013098 0.0001 0.000 292.29 55.35 0.95

*MM 0.0015122 0.0002 0.000 205.62 16.24 11

*MSF 0.0028219 0.0002 0.000 283.84 18.49 14

*MF 0.0030501 0.0002 0.000 308.46 20.66 8.1

ALP1 0.0343966 0.0003 0.000 17.47 52.63 1.2

*2Q1 0.0357064 0.0004 0.000 151.24 37.41 2.2

*SIG1 0.0359087 0.0005 0.000 50.76 25.21 6.2

*Q1 0.0372185 0.0144 0.000 261.97 0.90 3.7e+03

RHO1 0.0374209 0.0003 0.000 93.56 44.60 2

*O1 0.0387307 0.0538 0.000 317.01 0.25 5.9e+04

*TAU1 0.0389588 0.0006 0.000 122.18 36.70 2.1

BET1 0.0400404 0.0002 0.000 50.00 75.43 0.7

*NO1 0.0402686 0.0011 0.000 129.25 21.04 4.6

CHI1 0.0404710 0.0003 0.000 54.08 42.06 1.8

*PI1 0.0414385 0.0005 0.000 16.12 32.36 3.9

*P1 0.0415526 0.0230 0.000 57.03 0.73 8.4e+03

*S1 0.0416667 0.0007 0.000 176.72 32.95 2.9

*K1 0.0417807 0.0702 0.000 57.49 0.19 8.4e+04

*PSI1 0.0418948 0.0008 0.000 78.86 18.11 11

*PHI1 0.0420089 0.0007 0.000 183.05 19.20 7.3

*THE1 0.0430905 0.0004 0.000 234.63 37.47 3.2

*J1 0.0432929 0.0005 0.000 182.36 24.66 6

*SO1 0.0446027 0.0007 0.000 211.75 16.08 12

*OO1 0.0448308 0.0003 0.000 255.74 33.08 3.3

*UPS1 0.0463430 0.0004 0.000 309.46 26.87 4.7

OQ2 0.0759749 0.0008 0.001 166.33 54.91 0.86

*EPS2 0.0761773 0.0025 0.001 137.10 20.19 8.9

*2N2 0.0774871 0.0291 0.001 31.38 1.66 1e+03

*MU2 0.0776895 0.0084 0.001 169.39 7.11 81

*N2 0.0789992 0.2227 0.001 52.09 0.26 4.7e+04

NU2 0.0792016 0.0010 0.001 75.75 55.67 1.2

*GAM2 0.0803090 0.0019 0.001 149.09 29.66 4

*H1 0.0803973 0.0021 0.001 62.03 26.92 4.4

*M2 0.0805114 1.0566 0.001 68.69 0.06 1.1e+06

*H2 0.0806255 0.0027 0.001 178.75 20.15 5.6

*MKS2 0.0807396 0.0020 0.001 261.70 27.84 5.4

*LDA2 0.0818212 0.0022 0.001 75.48 28.52 5.2

*L2 0.0820236 0.0038 0.001 51.96 11.36 28

*T2 0.0832193 0.0017 0.001 88.60 33.53 2.8

*S2 0.0833333 0.3597 0.001 95.96 0.16 1.4e+05

*R2 0.0834474 0.0021 0.001 95.82 20.34 7.5

*K2 0.0835615 0.0977 0.001 89.43 0.42 1.2e+04

*MSN2 0.0848455 0.0022 0.001 288.67 23.60 3.6

ETA2 0.0850736 0.0003 0.001 357.26 107.97 0.46

*MO3 0.1192421 0.0004 0.000 255.52 24.21 4.8

*M3 0.1207671 0.0007 0.000 288.79 14.68 17

SO3 0.1220640 0.0001 0.000 259.25 69.21 0.81

*MK3 0.1222921 0.0009 0.000 268.12 9.87 31

*SK3 0.1251141 0.0006 0.000 342.53 14.84 13

*MN4 0.1595106 0.0059 0.001 264.37 6.65 98

*M4 0.1610228 0.0209 0.001 257.26 1.73 8.4e+02

*SN4 0.1623326 0.0019 0.001 343.91 20.42 5.6

*MS4 0.1638447 0.0107 0.001 312.28 3.47 2.8e+02

*MK4 0.1640729 0.0029 0.001 310.57 10.03 31

*S4 0.1666667 0.0015 0.001 23.29 23.14 5.8

*SK4 0.1668948 0.0009 0.000 25.94 33.96 3.4

*2MK5 0.2028035 0.0008 0.000 257.60 14.41 18

2SK5 0.2084474 0.0002 0.000 353.73 63.45 0.94

*2MN6 0.2400221 0.0022 0.000 321.39 9.85 37

*M6 0.2415342 0.0040 0.000 347.45 4.94 1.3e+02

*2MS6 0.2443561 0.0040 0.000 14.61 4.83 1.3e+02

*2MK6 0.2445843 0.0010 0.000 1.21 15.93 13

*2SM6 0.2471781 0.0010 0.000 21.28 20.35 8.7

*MSK6 0.2474062 0.0005 0.000 16.85 30.45 3.8

3MK7 0.2833149 0.0003 0.000 82.05 58.90 1.3

*M8 0.3220456 0.0018 0.001 175.81 16.58 11
